# Lifting the *cloche*: Jeroen Bakkers interviews Didier Stainier

**DOI:** 10.1242/dmm.050147

**Published:** 2023-03-28

**Authors:** Didier Y. R. Stainier, Jeroen Bakkers

**Affiliations:** ^1^ Max Planck Institute for Heart and Lung Research, Department of Developmental Genetics, Bad Nauheim 61231, Germany; ^2^ Hubrecht Institute-KNAW and Utrecht University Medical Center, Utrecht 3584 CT, The Netherlands; ^3^ Department of Pediatric Cardiology, Division of Pediatrics, University Medical Center Utrecht, Utrecht 3584 EA, The Netherlands

## Abstract

Didier Stainier is Director of the Department of Developmental Genetics at the Max Planck Institute for Heart and Lung Research in Bad Nauheim, Germany. He became acquainted with the zebrafish model as a PhD student in Walter Gilbert's lab at Harvard, which motivated him to champion the use of this powerful model organism to study heart development as a postdoctoral fellow with Mark Fishman at Massachusetts General Hospital. Although his scientific focus has expanded significantly since then, zebrafish modelling and heart development and regeneration remain key topics in his research. The developmental biology and zebrafish modelling communities have embraced him as an inspiring mentor and advocate for basic research.

Jeroen Bakkers is a group leader at the Hubrecht Institute for Developmental Biology and Stem Cell Research and Professor of Molecular Cardiogenetics at the University Medical Center Utrecht, The Netherlands. Jeroen did hid PhD with Herman Spaink at Leiden University, The Netherlands. A short visit to Massachusetts Institute of Technology during his doctoral training introduced him to the zebrafish model, which he applied to his PhD project. Zebrafish development remained the focus of his career, including during his postdoctoral training in the lab of Matthias Hammerschmidt at the Max Planck Institute of Immunology and Epigenetics in Freiburg and in his own lab at the Hubrecht Institute, where his group uses this powerful model organism to investigate cardiac development, disease and regeneration.

Jeroen and Didier met up at a recent conference to talk about their shared interest in cardiac regeneration, a zebrafish mutant with a curious name and Didier's commitment to mentorship.


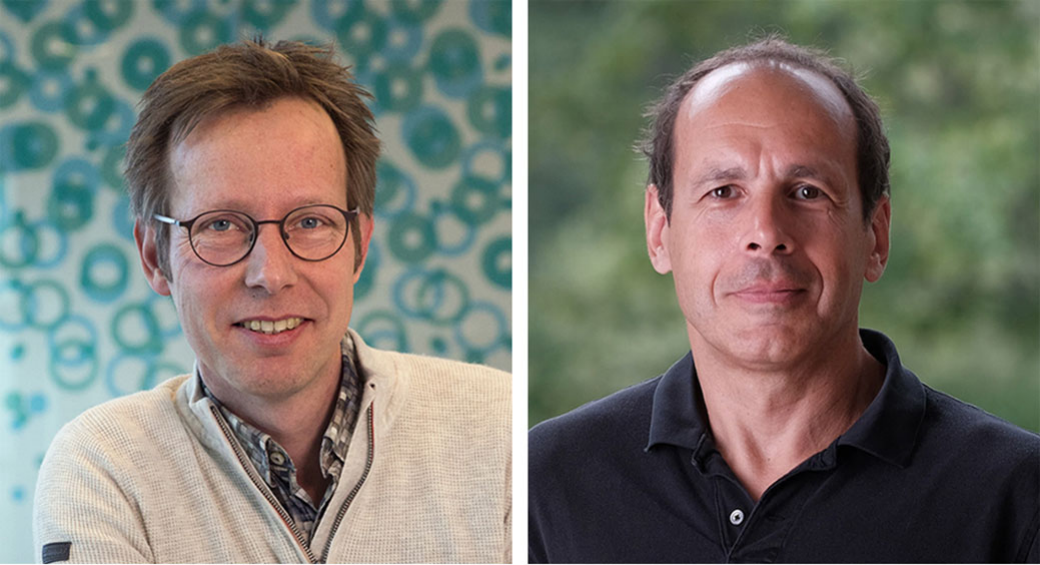


**Jeroen Bakkers (left) and Didier Stainier (right)**

**Jeroen Bakkers:** We met for the first time when I was a postdoc in Matthias’ [Hammerschmidt] lab and you came to give a talk [at the Max Planck Institute for Immunology and Epigenetics] in Freiburg.

**Didier Stainier:** Yes, I remember. This must have been about 20 years ago!

**Jeroen:** Indeed, it was in 2002. At the time, I had not yet started my transition to [the Hubrecht Institute in] Utrecht. We talked about interesting gene expression patterns that I wasn't sure were related to the heart or not, and we have been following each other's career ever since. Once I started my lab, I worked on the BMP pathway mutants and you were working on cardiac mutants from the ‘Boston’ screen [a large-scale mutagenesis screen of the zebrafish genome performed in Wolfgang Driever's lab at Harvard in the early 1990s; the cardiac mutant phenotypes are described in Stainier et al. (1996)]. This screen influenced quite a bit of your work. Was there a particular mutant or group of mutants that drove your research direction?

**Didier:** As a postdoc in Mark's group, I participated in this forward genetic screening effort. Of all the interesting mutants we identified, one I found particularly fascinating was *cloche* [which got its name from the characteristic shape of the mutant heart]. These fish lack endothelial cells, including endocardial cells, but a heart is formed (Stainier et al., 1995). Actually, we identified this phenotype before the screen, caused by a background mutation in zebrafish we had obtained from Indonesia. This naturally occurring mutation caused a striking phenotype, which boded well for the screen in which another allele (*clo^m378^*) was identified (Stainier et al., 1996). Although the screen produced many striking cardiac phenotypes, such as cardia bifida, fibrillation or valve defects, I wanted to stay focused. And several of these cardiac phenotypes were also identified in the Tübingen screen [led by Christiane Nüsslein-Volhard; see Haffter et al. (1996)]. When one has a very large collection of mutants, it's too easy to get distracted! And at that time, getting from mutant phenotype to gene was not easy.

**Jeroen:** Indeed, positional cloning could take years…

**Didier:** And some mutants proved harder to clone than others. Such work required a dedicated person. Once I started my own lab [at the University of California San Francisco (UCSF)], and as we progressed with cloning the more approachable mutants, we learned not only about their biology, but we also refined the techniques and became experts in positional cloning. These advances further empowered us to find the *cloche* gene. This project seemed too risky for a graduate student, but, luckily, Suk-Won Jin, a postdoc who had previously done some heroic positional cloning in *Caenorhabditis elegans*, decided to take this on.

**Jeroen:** He took on the challenge! The *cloche* locus is in a telomeric region and thus difficult to get hold of.

**Didier:** Exactly, no bacterial artificial chromosomes were available for that region, there was no annotation, and of course this work was being done before next-generation sequencing methods were available. We were mapping a huge number of embryos and, for a while, all we would talk about in lab meetings was positional cloning techniques!

**Jeroen:** Did it help at all that so little was known and you needed to discover other things about this mutant?

**Didier:** In a way, yes. Technically, it helped that we could learn from the other mutants and became experts in positional cloning, so that we could tackle this ‘unclonable’ one. Of course, when you are working on a mutant, it's not enough to just map the gene, you also want and need to get to the biology. For example, Leon Parker, a postdoc in the lab, performed transplant experiments to determine whether the various phenotypes were cell autonomous. The findings were very interesting (Parker and Stainier, 1999), but told us that the picture was also somewhat complicated.

“Of course, when you are working on a mutant, it's not enough to just map the gene, you also want and need to get to the biology.”

**Jeroen:** The paper describing the cloning of the *cloche* gene came out not that long ago, right?

**Didier:** Yes, we finally published the paper 6* *years ago (Reischauer et al., 2016). An extremely long gap between when we first observed the mutant phenotype and when we cloned the gene [which we named *npas4l* due to its sequence homology to *npas4* genes, which encode transcription factors involved in neuronal development].

**Jeroen:** There was also some controversy, some claims…

**Didier:** Yes, a previous paper reported that the *cloche* phenotype was caused by a mutation in another gene [*lysocardiolipin acyltransferase* (*lycat*; also known as *lclat1*) (Xiong et al., 2008)], although those claims were not definitive. We persisted, and this gave more space to postdocs in my lab to develop their own projects. We also pursued cardia bifida mutants, which led us to start working more on endoderm development.

**Jeroen:** …and the roles of various genes in these two tissues [the endoderm and mesoderm]?

**Didier:** Yes, especially as lab members like Debbie [Yelon] set up their own labs working on heart development, I felt that heart research was in very good hands. I could then focus on other organs and tissues.

**Jeroen:** Going back to the beginning and career choices, did you always want to be a scientist?

**Didier:** Yes, I did and I would say that it was a combination of environment and genetics for me. But mostly environment [laughs].

**Jeroen:** What was it about your environment?

**Didier:** There were several medical doctors and pharmacists on my father's side of the family, so family reunions were all about medicine and pharmacy. And my mother is a biologist who was initially doing research and later became a biology teacher. I didn't want to be a physician, so naturally gravitated towards becoming a scientist… I moved from Belgium to the USA because I wanted to do research as part of my undergraduate studies, and undergraduate research was not possible in Belgium at the time. So I transferred to Brandeis University and worked in Eric Selsing's lab, an immunology lab. I had never worked in a lab before, but I was given complete freedom, doing Southern blots over and over again looking for double-stranded DNA breaks in specific immune genes. Of course postdocs and students helped me get started, but it was amazing being able to essentially do my own research.

**Jeroen:** You then did your PhD in Walter Gilbert's lab. How was that?

**Didier:** Wally's lab was in some ways a very similar environment. Of course, it was bigger and we worked on many different topics, but we also had a lot of freedom. The project I started with two postdocs in the lab was to raise monoclonal antibodies against cell surface antigens that would allow us to look at subsets of embryonic neurons in the mouse, hoping to find molecules involved in axon guidance and target recognition. But there were other people working on exon–intron gene structure, DNA-sequencing technologies and some cellular immunology. Again, people were very independent in terms of the day-to-day experiments. Wally would still come to the lab in the evenings, sit at the microscope and look at mouse brain preparations, and this was after the [Nobel] Prize. It was quite amazing. Obviously, once the Human Genome Project started, he got very busy and we didn't see him much. But he is probably the smartest scientist I've met and remains a big influence. Wally was also running the ‘Junior Fellows’ Program at Harvard until recently. It's a unique program for PhD graduates where the Harvard Society of Fellows pays their salary and the fellow can work anywhere in Boston. Mohamed [El-Brolosy], who was a PhD student in my lab, is now one of these Junior Fellows. Part of the program is the weekly Monday night networking dinners. I happened to attend one of these dinners with Mohamed, and Wally was also there. At 90 years of age, he is still amazingly sharp and passionate about science and photography. I was lucky that Mark [Fishman]’s lab also gave me complete freedom within the broad remit of the lab. So I never worked with a micromanager and have been fortunate enough [in my own lab] at UCSF and later in Germany to provide an environment in which people can do their own things and build their own projects and grow as scientists, take risks, hit roadblocks and overcome them. I believe that one can grow as a scientist in many different kinds of environment, but I think that learning how to troubleshoot by oneself is key to this process.

“[I] have been fortunate enough [in my own lab] at UCSF and later in Germany to provide an environment in which people can do their own things and build their own projects and grow as scientists, take risks, hit roadblocks and overcome them.”

**Jeroen:** Switching gears to microscopy now…it has always been very important in your work, and, of course, zebrafish are really well suited for it. But you have always looked for new boundaries.

**Didier:** Yes. A lot of the credit goes to Jan Huisken [former postdoc and pioneering microscopist, currently an Alexander von Humboldt professor at the University of Göttingen]. We were always using the latest technologies, but it wasn't until Jan joined the lab that we started developing new microscopy technology and using it. Jan did his PhD with Ernst Stelzer, where he helped develop light-sheet microscopy. The way Jan tells the story [of how he came to Didier's lab at UCSF], is that he was looking for the most challenging organ to image [laughs]…

**Jeroen:** Which is the beating heart [laughs]!

**Didier:** Which is the beating heart. Of course, to image it, it needs to be accessible. Jan had been exposed to zebrafish at the European Molecular Biology Laboratory [during his PhD], so we were really fortunate that he joined the lab. He helped people in the lab with so many different projects – looking at the formation of the cardiac valves, revealing interesting patterns in the ventricle, imaging endodermal cell migration, examining endothelial cell sprouting… He is such a community-oriented person, and he is still helping the community at large by making his ‘travelling’ microscopes [the Huisken lab has developed the Flamingo microscope, which can be moved and remotely configured, increasing access to advanced imaging regardless of other infrastructure].

**Jeroen:** Are there any new developments in microscopy that you are excited about? Things that would be good for the zebrafish field or for cardiac research?

**Didier:** As you saw yourself when you came to visit, we now have many high-end microscopes and a dedicated staff scientist to run our microscopy core. Of course, getting better resolution remains a major challenge. There are microscopes that give you exceptional resolution, but depth is an issue. One can see superficial structures really well, but not deep into a living sample. So being able to look deep at high resolution is still of course a big challenge. This is also the case in the area of zebrafish adult heart regeneration. As you know, imaging zebrafish adult heart regeneration is the next challenge.

“[…] imaging zebrafish adult heart regeneration is the next challenge.”

**Jeroen:** This was going to be my next question! How did you get involved in heart regeneration research and in which direction do you think this is going in the next 10 years?

**Didier:** It's often about trying to give space to postdocs in the lab so that they can develop their own project and take it with them when they start their own labs. Because it's easier for me to start something new than it is for new PIs. So although heart regeneration is not a new field, it was new for us. Ken [Poss] of course published the initial paper [on cardiac regeneration in zebrafish (Poss et al., 2002)]. We came into this topic from our work on the nitroreductase system [for targeted tissue ablation, which Didier's lab and others developed]. Silvia Curado started this work in the lab and observed [heart regeneration upon nitroreductase-mediated ablation] in embryos and larvae (Curado et al., 2007). Other labs were working on mouse neonatal hearts and claimed that what they observed was not really regeneration, but was due to the normal growth process. One could make the same argument for what we observed in zebrafish embryos and larvae. To prove that it was indeed regeneration, we needed to work in adult fish. As far as where I think that the field will be in 10 years, I think we will have a pretty good understanding of what the various cell types are doing, both in terms of their individual gene expression profiles, but also how they affect other cell types in the regenerating heart.

We will have a more holistic view of the cellular and molecular interactions at different stages of the process. As you know very well, the field has been very focused on cardiomyocyte proliferation. But now it's becoming clear that revascularisation, the immune system, fibroblasts and innervation all come into play. The field is still looking at these processes on a daily time scale but we need to study it at the hourly scale! Regeneration is a lot like development, but more complicated. Heart development starts with a few cells, and involves growth and patterning. In regeneration, the tissue is already in a complex environment with multiple cell types that respond to injury and interact in various ways. Some of my former trainees who started their own lab recently have been under great pressure to incorporate mouse work in their studies, so I'm also hoping that there will be enough interest from researchers who use mammalian regeneration models to support us [zebrafish researchers] because there is so much to gain from a very detailed understanding of how the zebrafish regenerates its heart.

“[…] there is so much to gain from a very detailed understanding of how the zebrafish regenerates its heart.”

**Jeroen:** In a natural, genetically non-perturbed situation?

**Didier:** Exactly. I'm preaching to the converted [laughs]! Unfortunately, funders don't always see this point and zebrafish work doesn't get enough support. Of course, work in mouse, and beyond, needs to happen to translate findings from fish to mammals. We also need to better understand the immune response [to cardiac injury], revascularisation, the role of innervation, fibroblasts… It is now clear that multiple types of macrophages and fibroblasts are involved; maybe we will soon discover that there is greater cardiomyocyte heterogeneity than anticipated. There is definitely still a lot of work to do.

**Jeroen:** So what kinds of challenges do you think need to be overcome? Technical? Adult fish are not transparent, so we cannot image their hearts.

**Didier:** As you showed [in your talk at this conference], you can do a lot with [*ex vivo* cardiac] slices in culture. Of course, we really want to do *in vivo* imaging. Maybe in 10 years we will have a tiny camera inside the heart. Think about how far we have come with optogenetics; maybe someday we will be able to image cell–cell interactions in the heart in real time.

**Jeroen:** These techniques will allow us to look at the early events [post cardiac injury; as described in Honkoop et al. (2021)], but we also don't understand the late events – scar resolution, migration, other important aspects that determine successful regeneration.

**Didier:** I agree. Some people in the lab have done work in mouse, and it's not so trivial. As an intermediate of sorts, the spiny mouse could be a viable model. Overall, this area will remain an exciting one for many years to come; cardioid-based approaches will also likely gain preclinical power, but these will need validation in living systems.

**Jeroen:** We could resume this conversation in 10 years and we can then discuss what the next big things are.

**Didier:** Yes, although in 10 years, I will have to think about retiring from the Max Planck [laughs].

**Jeroen:** We can then catch up [laughs]! The other topic I wanted to mention is transcriptional adaptation, something you talked about in your talk [at the 15th Zebrafish Disease Models Society meeting]. You have been working on this for a while. How do you think this would influence the field of genetics?

**Didier:** I never thought I'd be working on a topic like this! But because I most enjoy learning something new every day, I now find myself thinking about topics like transcriptional pausing, small RNA biogenesis, antisense RNAs… Because I ‘grew up’ in Wally's molecular biology lab, I never really abandoned the RNA world. To answer your question, there is evidence that this process is relevant to human genetics, and our work is composed of three main lines: understanding the underlying mechanisms, how relevant this is to humans, and whether it has therapeutic potential. For the first line, the mechanistic aspect, we are trying to identify the ‘simplest’ model organism that displays this phenomenon, and so we are now looking at *Neurospora* because they have many introns as well as the RNA interference machinery, and many other key components. For the therapeutic angle, for example, in sickle cell anaemia, upregulating the expression of foetal haemoglobin helps the patient. A possible avenue would then be to introduce a mutation in the sickle cell gene that would lead to mutant mRNA degradation, and this would in turn upregulate the expression of foetal haemoglobin.

**Jeroen:** So this would use the basic concepts of transcriptional adaptation to treat patients?

**Didier:** Exactly. Of course, there are already approaches that upregulate foetal haemoglobin that don't rely on transcriptional adaptation, but we are trying to find a good site in the beta haemoglobin gene to introduce the mutation, to see if the degradation of the transcript with the mutation would indeed trigger the expression of the foetal gene.

**Jeroen:** Are you trying this in mouse models?

**Didier:** We first tried in cell lines. One problem is that most cells do not express globins, so we had to identify a precursor cell line that can differentiate into globin-producing cells. If our experiments work in this line, then we will need to bring them into the mouse. The therapeutic aspect is one of the ultimate goals of course, and we are also looking for collaborators in the human genetics field who could provide us with patient samples that carry mutations that cause mRNA degradation. I'm actively engaging with this community, presenting the work at human genetics meetings to establish such collaborations and analyse tissue samples. Of course, tissues are composed of multiple cell types, and a gene of interest may be upregulated in one cell type but downregulated in the other. So this work will be challenging and it is keeping us busy.

**Jeroen:** And the mechanistic aspect means you are using different models – mouse, *C. elegans*, yeast and *Neurospora*.

**Didier:** And mammalian cells in culture. We were hoping that *C. elegans* would allow us to do forward genetic screens, but the transgenerational effects (Jiang et al., 2022) make this more complicated than we had anticipated. When I give talks about this work, different people have different reactions, some of them asking “Why are you making things so complicated?” [both laugh].

**Jeroen:** That is a strange question to ask. Biology is complicated! This is why it is so nice to study

**Didier:** Exactly! We are not going to solve it tomorrow. We are adding another layer of complexity to the genotype–phenotype relationship.

**Jeroen:** You run so many different projects. I always wanted to know how you keep track?

**Didier:** I think it has to do with having the right people in the lab. The lab really only works on three main topics: cardiac development, cardiac regeneration and transcriptional adaptation. We have monthly brainstorming meetings and regular lab meetings. There is a lot of interaction when a new member joins the lab to define the project and get it going. Whenever someone presents in the lab meeting, they send me their data so that I can review them after the meeting to get a solid overview and ensure I remember the results correctly. Of course, I expect the lab members to become experts in their specific areas. It's about building a community and subgroups within this community that are up to date with the literature. And of course, when it's time to write a paper, we pull together. When I interview candidates, I keep everything transparent – the size of the lab, how it's organised… Because I want them to really think whether this is the right place for them.

**Jeroen:** When the paper is being written, there is more active supervision and involvement. Is this also part of your mentoring approach?

**Didier:** I think that the most important thing to realise is that everyone is different and you need to get a good sense of how much freedom they need, when they need encouragement and what kind. People are trying to grow as scientists, they come with different strengths and from different backgrounds, so it's about tailoring the interactions. Mentoring is not just about my relationship with them, it's also about peer interactions and the relationships between senior and junior members. So it's about having the right people in the lab. I think it's so important to provide the right environment.

**Jeroen:** And this starts with selecting the right people?

**Didier:** Yes, in a way, it's a positive-feedback loop. When new team members see this kind of environment, they feel more generous as well.

**Jeroen:** And when your postdocs are ready to set up their own labs, how do you help them?

**Didier:** Everyone knows that they can take anything they want with them, unless it's data or reagents that were generated by someone else and, in this case, they need to get that person's permission. Of course, we are not going to stop working on a broad topic, like cardiac regeneration, if someone wishes to focus on it in their own lab. But we are always transparent about what we do and what former lab members do, so the incoming student or postdoc is fully aware. In the long run, this attitude is beneficial to the lab because people know that their work will be protected. In a way, this is facilitated because of the [Max Planck] funding and we don't have to struggle with grant applications. It's an unusual, and very privileged model. I'm very lucky. There are generously funded positions elsewhere, but I think that only Germany offers this level of scientific freedom.
